# Robot-Assisted Laparoscopic Radical Prostatectomy in a Patient With Ectopic Ureter

**DOI:** 10.7759/cureus.93909

**Published:** 2025-10-05

**Authors:** Junji Yatsuda, Hideaki Nishizawa, Toshiki Anami, Kurahashi Ryoma, Murakami Yoji, Tomomi Kamba

**Affiliations:** 1 Department of Urology, Graduate School of Medical Sciences, Kumamoto University, Kumamoto, JPN

**Keywords:** ectopic ureter, positive surgical margin, prostate cancer, rarp, ureteral duplication

## Abstract

An ectopic ureter is a rare male anomaly. We report a case of robot-assisted radical prostatectomy for prostate cancer in a patient with an ectopic ureter and a duplicated collecting system. A 71-year-old man was diagnosed with prostate cancer. Preoperative evaluation identified a left ectopic ureter opening into the bladder neck. During robot-assisted radical prostatectomy, an attempt to preserve the ectopic orifice resulted in an extensive positive surgical margin. The patient subsequently developed biochemical recurrence and was treated with androgen deprivation therapy and salvage radiotherapy. His prostate-specific antigen level is now undetectable. Preoperative diagnosis of an ectopic ureter is crucial for surgical planning to avoid positive margins and complications. This anomaly should be considered if an unexpected tubular structure is encountered intraoperatively or if unexpected urinary leakage occurs postoperatively.

## Introduction

An ectopic ureter is a rare congenital anomaly that predominantly affects females, while in males it typically drains into the prostatic urethra, seminal vesicle, or vas deferens [1]. Radical prostatectomy remains the gold standard for the treatment of prostate cancer, and robot-assisted surgery, in particular, is widely utilized in numerous countries and institutions due to its precise maneuverability and other advantages. In patients with an ectopic ureter, prostatectomy can lead to severe complications if the condition is not diagnosed and addressed preoperatively or intraoperatively, as this may leave a patent ureter in the surgical field. Herein, we report a case of robot-assisted laparoscopic radical prostatectomy (RARP) performed on a patient with an ectopic ureter opening into the bladder neck.

## Case presentation

**Figure 1:**
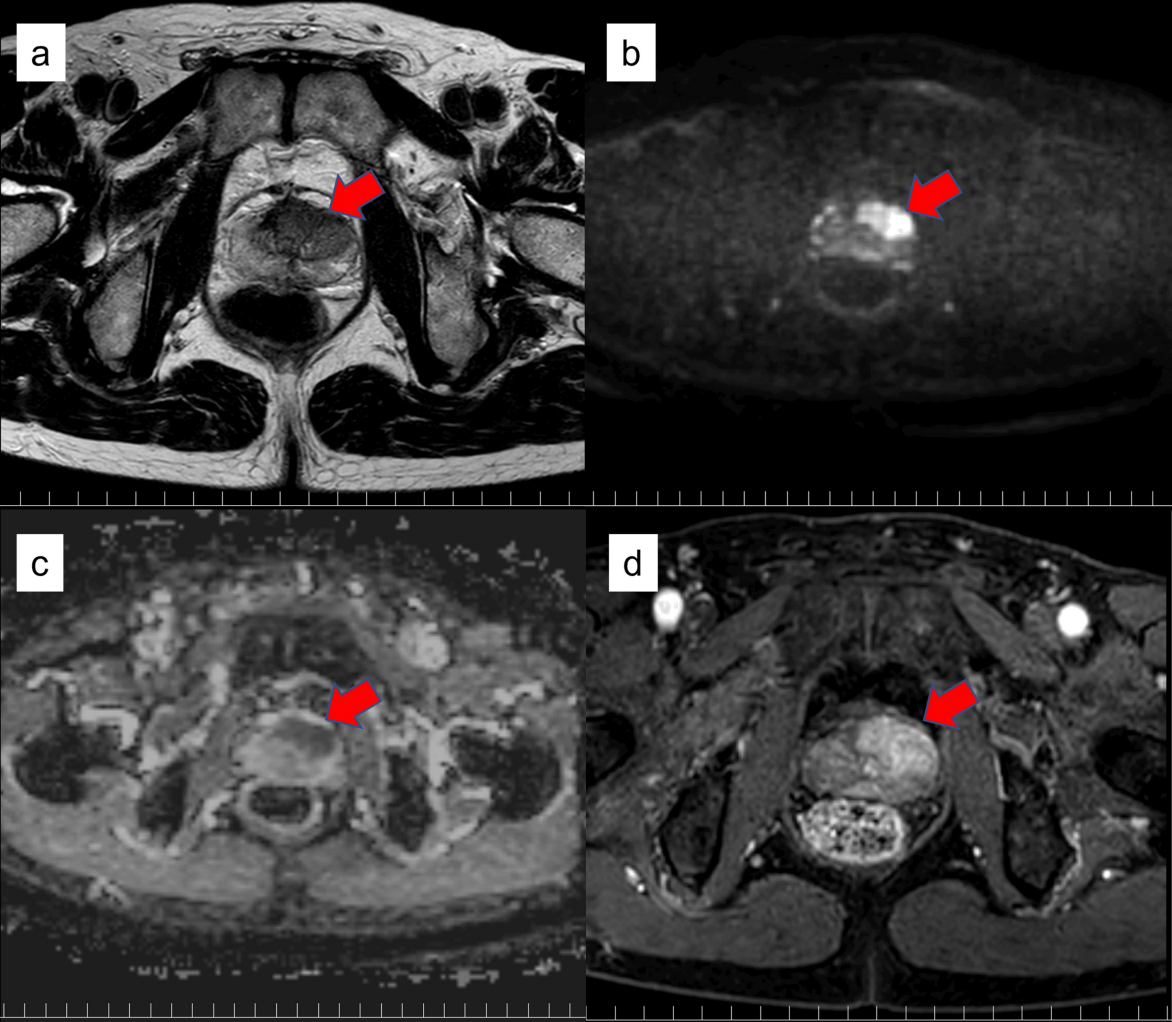
Preoperative multiparametric magnetic resonance imaging of the prostate An extensive lesion suspicious for malignancy, indicated by red arrows, was identified in the ventral aspect of the left prostatic lobe (a: T2-weighted image; b: diffusion-weighted image; c: apparent diffusion coefficient map; and d: contrast-enhanced image).

The patient was a 71-year-old man with no significant medical history. In February 2022, a routine health check-up revealed an elevated prostate-specific antigen (PSA) level of 35.83 ng/mL, for which he consulted a physician. A prostate magnetic resonance imaging (MRI) was performed, showing findings suggestive of prostate cancer with extraprostatic extension in the left lobe (Figure 1). Consequently, a prostate biopsy was conducted in March of the same year. The biopsy detected adenocarcinoma with a Gleason score of 4+3=7 in five cores from the left lobe. He was subsequently referred to our department for surgical management. Staging with contrast-enhanced computed tomography (CT) and bone scintigraphy showed no evidence of distant metastasis. However, bilateral complete ureteral duplication was incidentally suspected, prompting a detailed investigation.

**Figure 2:**
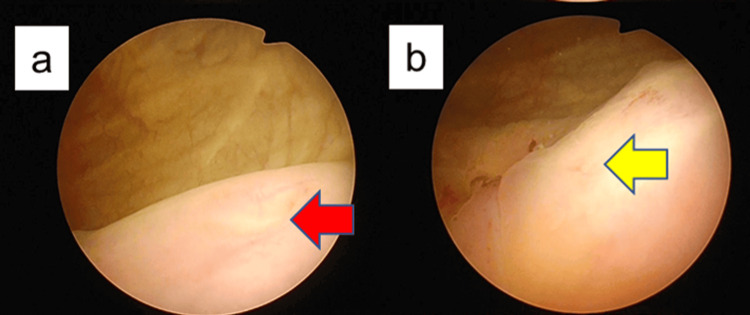
Preoperative cystoscopy Red arrowhead indicates the normal left ureteral orifice (a: trigone of the urinary bladder), and yellow arrowhead indicates the ectopic left ureteral orifice (b: bladder neck).

**Figure 3:**
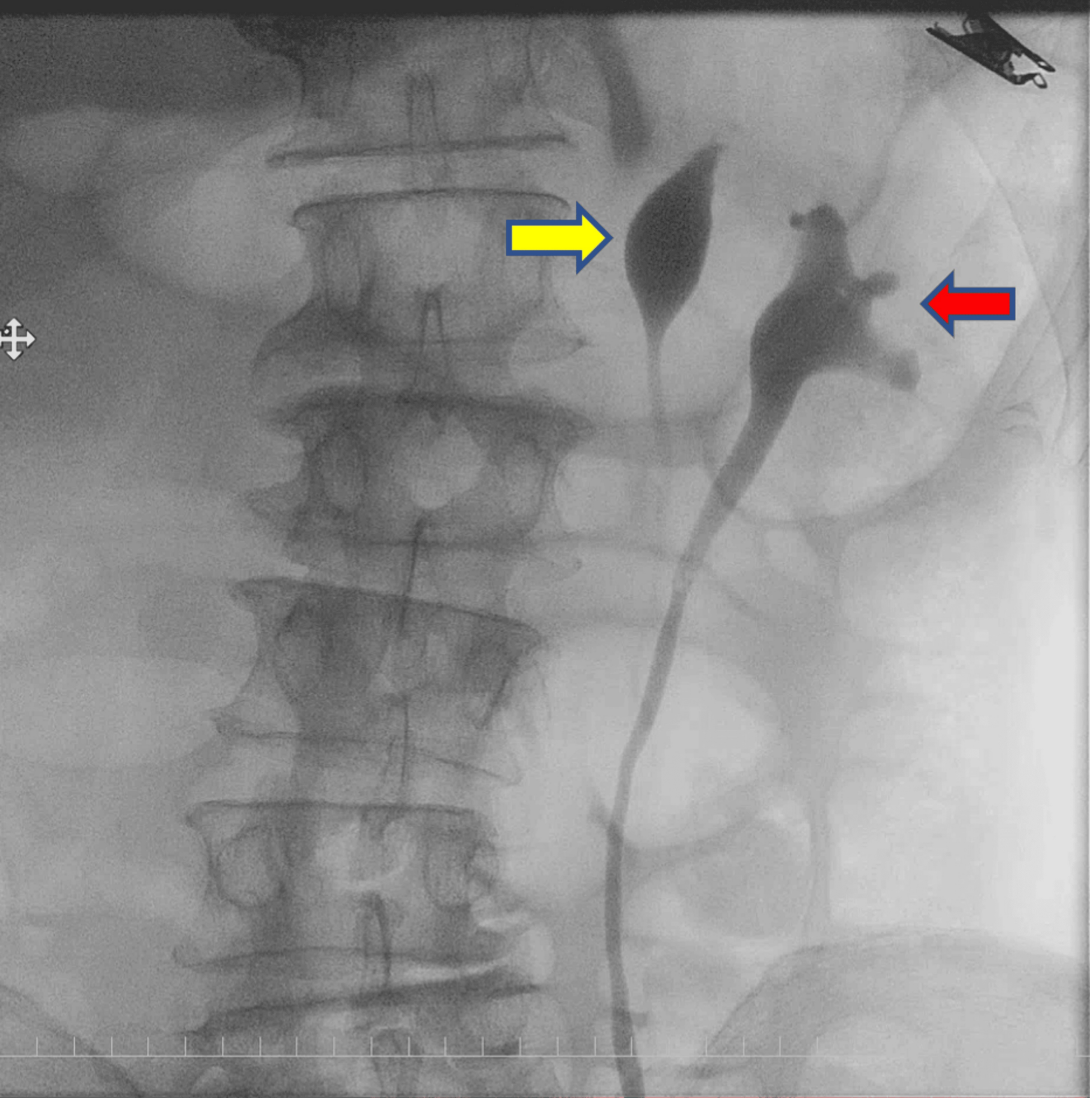
Preoperative retrograde pyelography Red arrowhead indicates the left lower-pole renal pelvis (normal kidney), and yellow arrowhead indicates the left upper-pole renal pelvis (atrophic kidney).

Cystoscopy identified two right ureteral orifices, one in the orthotopic position and the other in the vesical trigone near the internal urethral orifice. On the left side, only the orthotopic orifice was initially visualized. Suspecting an additional ectopic orifice on the left, a further examination was performed under anesthesia. This revealed an ectopic left ureteral orifice located near the bladder neck, in close proximity to the prostatic urethra (Figure 2). Retrograde pyelography performed through each of the two left orifices opacified two separate renal pelves, confirming complete duplication (Figure 3).

**Figure 4:**
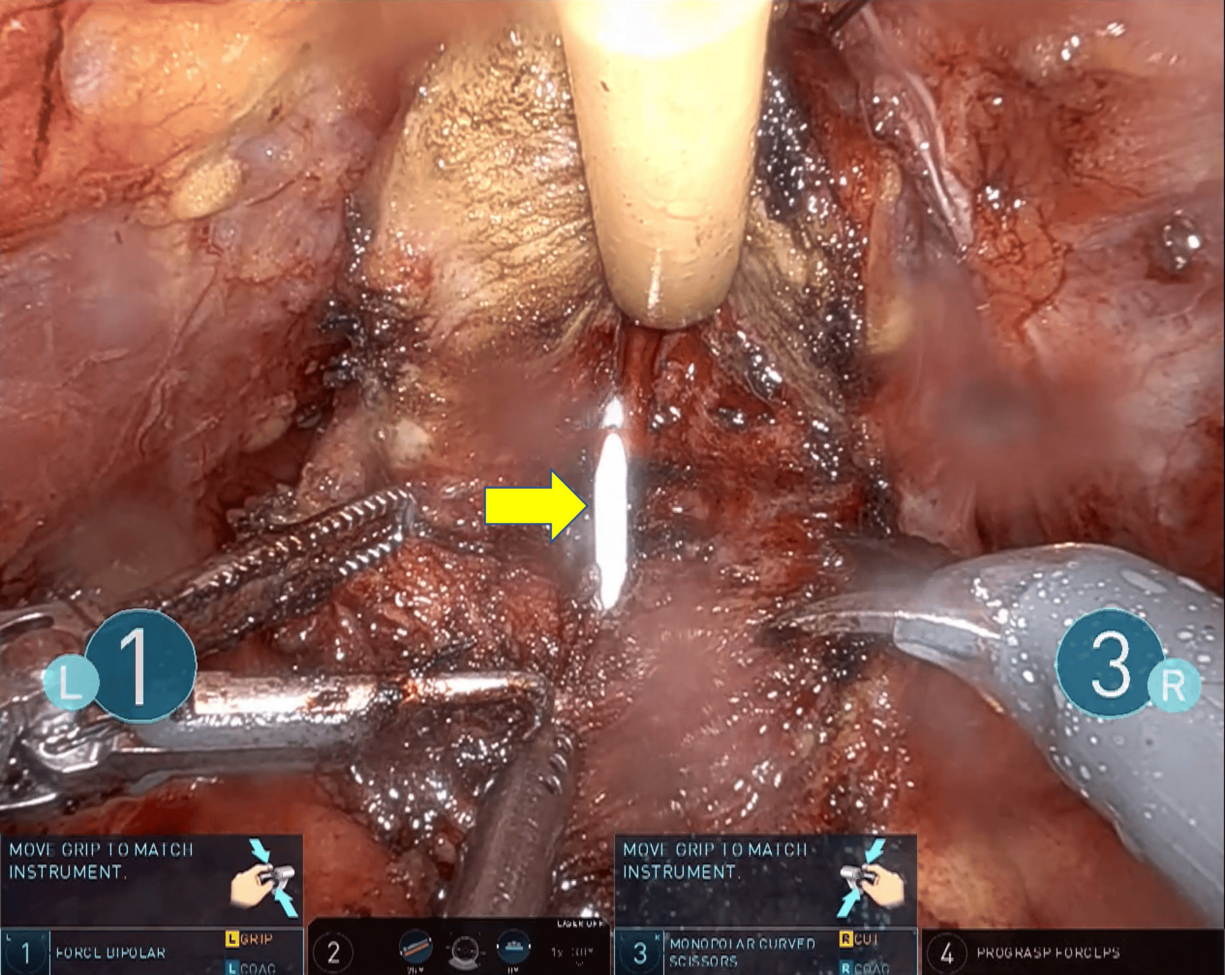
A ureteral stent was placed during bladder neck dissection The yellow arrowhead indicates a ureteral stent inserted transurethrally into the ectopic ureteral orifice.

Based on these findings, a decision was made to proceed with RARP following the preoperative placement of ureteral stents. Following the preoperative placement of a stent in the left ectopic ureter, the patient underwent RARP with extended pelvic lymph node dissection. The total operative time was eight hours and one minute, with a console time of five hours and 31 minutes. The estimated blood loss was minimal at 20 mL. During the procedure, the indwelling ureteral stent was used as a landmark to guide the bladder neck dissection and avoid injury to the left ectopic ureteral orifice (Figure 4). However, in an effort to carefully preserve this orifice, the dissection plane inadvertently extended into the prostate itself. This resulted in an extensive positive surgical margin at the bladder neck on final pathology. The patient experienced immediate postoperative biochemical recurrence and was subsequently treated with androgen deprivation therapy and salvage radiation therapy. Currently, his PSA level remains at an undetectable level.

## Discussion

An ectopic ureter is a congenital anomaly in which the ureter inserts outside the vesical trigone. The incidence is reported to be one in 2,000 to 4,000 individuals, with a significant female predominance at a 7:1 ratio. In males, the ectopic ureteral orifice is always located proximal to the external sphincter, most commonly in the prostatic urethra (54%). Other sites of insertion include the seminal vesicle (28%), vas deferens (10%), and the ejaculatory duct (8%) [1]. In 80-85% of cases, an ectopic ureter is associated with a duplicated renal collecting system [2]. Generally, the orifice of an ectopic ureter originating from a duplicated system follows the Weigert-Meyer rule. In the present case, the presence of a duplicated ureter led to the diagnosis of an ectopic ureter. However, since ectopic orifices can also occur in a single ureteral system, preoperative diagnosis in such cases can be challenging.

RARP is widely established as the standard procedure for prostate cancer treatment due to its precision. However, performing RARP in patients with a coexisting ectopic ureter, a rare anatomical anomaly, poses a risk of postoperative complications such as intraperitoneal urine leakage [3-5] if the condition is not diagnosed preoperatively. Therefore, preoperative diagnosis is crucial for the surgical management of prostate cancer with a concomitant ectopic ureter. Although the ectopic ureter was identified on CT in this case, a report suggests that MRI is also effective for its detection [6]. In our case, the ectopic ureter opened into the bladder neck and was therefore preserved, but this resulted in a positive surgical margin. Given that the upper pole of the kidney on the affected side was atrophic, sacrificing the ureter might have been a better option from an oncological control perspective.

To the best of our knowledge, a literature review revealed 14 previously reported cases of radical prostatectomy in patients with an ectopic ureter (Table 1). Seven cases were diagnosed preoperatively, two intraoperatively, and five were identified postoperatively due to urinary leakage. No postoperative problems were reported in the preoperatively diagnosed cases, whereas all cases identified postoperatively required reoperation, underscoring the critical importance of preoperative diagnosis. The most common site of the ectopic orifice was the prostatic urethra [3,7-12] (seven cases), followed by the seminal vesicle [13-17] (five cases), the bladder neck [5,18] (two cases), and one case in which the location was unknown [4]. Surgical management included urinary tract reconstruction, such as ureteroureterostomy [4,5,17] in three cases, ureteroureterostomy and ureteroneocystostomy [3,8] in two cases, nephroureterectomy [11,13] in two cases (for non-functioning or hypoplastic kidneys), and simple ureteral ligation [7,10,12] in three cases. One case, identified six months postoperatively, was managed with selective arterial embolization of the kidney [9].

**Table 1:** Clinical characteristics of patients with prostate cancer and concurrent ectopic ureter undergoing radical prostatectomy POD, postoperative day; POM, postoperative month

Timing of the diagnosis of ectopic ureter	Authors	Diagnostic trigger	Management of ectopic ureter	Site of ectopic ureteral insertion
Preoperative	Hubosky et al. (2007) [7]	-	Ureteroureterostomy and ureteroneocystostomy	Prostatic urethra
Preoperative	Funahashi et al. (2007) [8]	-	Ureteroureterostomy	Prostatic urethra
Preoperative	Marien et al. (2008) [9]	-	Ureteroureterostomy	Prostatic urethra
Preoperative	Matsumoto et al. (2016) [14]	-	Ureteral ligation	Seminal vesicle
Preoperative	Nakai et al. (2009) [13]	-	Nephroureterectomy	Seminal vesicle
Preoperative	Miyago et al. (2012) [10]	-	Upper pole heminephrectomy	Prostatic urethra
Preoperative	Spinos et al. (2023) [15]	-	Ureteral ligation	Seminal vesicle
Preoperative	Present case	-	No treatment	Bladder neck
Intraoperative	Shariat et al. (2005) [16]	-	Nephroureterectomy	Seminal vesicle
Intraoperative	Singhal et al. (2015) [11]	-	Ureteral ligation	Prostatic urethra
POD 2	Didier et al. (2017) [6]	Persistent urinary leakage	Ureteroureterostomy and ureteroneocystostomy	Prostatic urethra
POD 3	Hampton et al. (2008) [4]	Persistent urinary leakage	Ureteroureterostomy	Unknown
POD 3	Polo-Alonso et al. (2022) [17]	Fever up	Ureteroneocystostomy	Seminal vesicle
POD 8	Ghazi et al. (2011) [5]	Persistent urinary leakage	Nephrostomy and ureteroureterostomy	Bladder neck
POD 15	Minh et al. (2013) [18]	Fever up	Nephrostomy and ureteral stenting	Bladder neck
POM 6	Petrella et al. (2020) [12]	Frank pain	Selective arterial embolization	Prostatic urethra

## Conclusions

We report a case of radical prostatectomy in a patient with prostate cancer and a concomitant ectopic ureter. Preoperative diagnosis of an ectopic ureter allows for careful planning of the surgical approach. A primary objective is to achieve complete tumor resection with negative surgical margins to ensure oncologic control. Concurrently, it is essential to consider the appropriate method for urinary tract reconstruction if it becomes necessary. This anomaly should be suspected if an unexpected tubular structure is encountered intraoperatively or if unexpected urinary leakage occurs postoperatively.
